# The Role of Gluten in the Development of Autoimmune Thyroid Diseases: A Narrative Review

**DOI:** 10.5812/ijem-153730

**Published:** 2024-07-30

**Authors:** Kimia Sadat Esfahani, Nastaran Asri, Mohadeseh Mahmoudi Ghehsareh, Mostafa Rezaei-Tavirani, Somayeh Jahani-Sherafat, Mohammad Rostami-Nejad

**Affiliations:** 1 Celiac Disease and Gluten Related Disorders Research Center, Research Institute for Gastroenterology and Liver Diseases, Shahid Beheshti University of Medical Sciences, Tehran, Iran; 2 Gastroenterology and Liver Diseases Research Center, Research Institute for Gastroenterology and Liver Diseases, Shahid Beheshti University of Medical Sciences, Tehran, Iran; 3 Proteomics Research Center, Faculty of Paramedical Sciences, Shahid Beheshti University of Medical Sciences, Tehran, Iran; 4 Laser Application in Medical Sciences Research Center, Shahid Beheshti University of Medical Sciences, Tehran, Iran

**Keywords:** Gluten, Autoimmune Thyroid Disease, Celiac Disease, Gluten-Free Diet

## Abstract

**Context:**

In recent decades, adverse reactions to gluten have increased, collectively known as gluten-related disorders (GRDs). The most prominent GRD is celiac disease (CD), a T-cell-mediated autoimmune-like disorder of the small intestine triggered by the ingestion of gluten proteins in genetically predisposed individuals. Celiac disease is often associated with various autoimmune and idiopathic conditions, including autoimmune thyroid disorders (AITDs). Autoimmune thyroid disorders result from immune system dysregulation and lead to an assault on the thyroid gland. This study aims to examine the potential effect of gluten consumption on the development of AITDs.

**Evidence Acquisition:**

A narrative literature review was conducted using the Google Scholar, Scopus, and PubMed electronic databases to identify studies investigating the role of gluten in the development of AITDs. Keywords such as "Gluten," "Autoimmune thyroid disease," and "Gluten-free diet" were used.

**Results:**

The involvement of gluten in the pathogenesis of autoimmune diseases is mediated through various mechanisms. Gluten may contribute to the expansion and progression of AITDs through mechanisms such as dysbiosis, leaky gut, and cross-reactivity. There is evidence that adherence to a gluten-free diet (GFD) may positively impact patients with AITDs, supporting the importance of personalized dietary strategies to mitigate risks associated with gluten intake.

**Conclusions:**

The findings suggest that dietary management, particularly strict adherence to a GFD, may be beneficial for individuals with both CD and AITDs. Emerging evidence highlights the importance of personalized dietary strategies to mitigate the risks associated with gluten intake. A deeper understanding of the gut-thyroid axis could lead to the development of innovative approaches in the management of autoimmune disorders.

## 1. Context

Gluten-containing grains have been incorporated into the human diet for over 10,000 years. Gluten is extensively utilized in the food industry. These proteins consist of gliadin and glutenin subunits and are characterized as prolamins due to the substantial presence of glutamine and proline amino acid residues in their primary structures (1). Over the last few decades, an increase in adverse reactions to gluten, termed gluten-related disorders (GRDs), has been observed due to widespread gluten exposure (2). Gluten-related disorders are categorized based on their primary pathological mechanisms as autoimmune disorders, such as celiac disease (CD), dermatitis herpetiformis (DH), and gluten ataxia (GA); allergic conditions, such as wheat allergy (WA); and non-autoimmune/non-allergic conditions, like non-celiac gluten sensitivity (NCGS) (3). Currently, adherence to a gluten-free diet (GFD) constitutes the sole treatment for GRDs (4). Celiac disease is the predominant GRD, and its incidence has been increasing in recent years (5, 6).

The primary toxic components of gluten are proteins from the gliadin family. Due to the high concentration of proline residues, gliadin is resistant to digestion by gastric, pancreatic, and intestinal proteases. As a result, long fragments of gliadin accumulate in the gut epithelium (7). The incomplete digestion of gliadin results in the formation of two crucial peptides: The 33-mer immunogenic peptide (pp.57 - 89), which triggers a strong adaptive immune response, and a 25-amino acid peptide (pp.31 - 55), known to stimulate interleukin-15 (IL-15) production in both enterocytes and dendritic cells. These peptides not only initiate adaptive immune responses but also activate innate immune pathways, leading to cytotoxic effects on the intestinal epithelium (8, 9).

Class-II human leukocyte antigens (HLA) molecules are recognized as genes that predispose individuals to various diseases driven by inappropriate immune responses. For instance, HLA-DQ2 and DQ8 are associated with CD. When present in individuals, these genes can interact with gluten peptides, triggering immune responses that contribute to the development of the disease (9).

Celiac disease is a T-cell-mediated autoimmune disorder of the small intestine, triggered by gluten ingestion in genetically predisposed individuals. It affects about 1% of the population (10). The high glutamine and proline content in gliadin peptides makes them resistant to enzymatic digestion, resulting in their incomplete breakdown in the gastrointestinal tract (11). These gliadin peptides cross enterocytes, and the tissue transglutaminase (tTG) enzyme deamidates them, which are then recognized by HLA-DQ2 or -DQ8 on antigen-presenting cells (APCs). These APCs present the toxic peptides to CD4+ T cells, which, upon activation, produce pro-inflammatory cytokines. T helper 1 (Th1) cytokines enhance the cytotoxicity of intraepithelial lymphocytes (IELs) and natural killer (NK) T cells, leading to enterocyte apoptosis through the Fas/Fas ligand (FasL) system or IL-15-induced perforin/granzyme and NKG2D-MICA signaling. T helper 2 (Th2) cytokines activate B cells, leading to their clonal expansion and differentiation into antibody-secreting plasma cells (anti-gliadin and anti-tTG) (12).

Celiac disease is usually accompanied by gastrointestinal symptoms like abdominal discomfort, bloating, diarrhea, and nausea. If untreated, it can lead to chronic conditions and non-gastrointestinal issues such as anemia, osteoporosis, fatigue, infertility, eczema, and refractory CD, which increases lymphoma risk (13). Celiac disease is frequently associated with various autoimmune and idiopathic conditions, such as autoimmune thyroid disorders (AITDs) (14). Diagnosing CD in patients with these comorbidities is crucial because a GFD can alleviate symptoms, prevent complications, and improve certain CD-associated conditions (15).

Thyroid disorders are common conditions affecting the thyroid gland, involving the production of insufficient or excessive thyroid hormones, which are crucial for growth and metabolism. While thyroid dysfunction is often identifiable and treatable, misdiagnosed or untreated conditions can lead to severe complications (16).

The breakdown of self-tolerance to thyroid antigens leads to the onset of AITDs, a process that can be triggered by various factors, including excessive exposure to thyroid antigens, exposure to environmental antigens resembling self-antigens, polyclonal immune activation, or idiotype cross-reaction of self-antigens (17). Autoimmune thyroid disorders are characterized by immune system dysregulation and are the most prevalent autoimmune disorders in humans (18). The spectrum of AITDs includes Graves’ disease (GD), presenting as hyperthyroidism, Hashimoto’s thyroiditis (HT), and atrophic thyroiditis (AT), presenting as hypothyroidism (19, 20).

It is widely accepted that AITDs are multifactorial in nature, arising from a sophisticated interplay among genetic predispositions, hormonal fluctuations, and environmental factors (17). Environmental factors that may contribute to the development of AITDs include the amount of iodine intake, consumption of gluten and alcohol, deficiencies in selenium, iron, zinc, and vitamin D, high levels of stress, pregnancy, and the use of key immune modulators such as interferon, ipilimumab, and alemtuzumab (21). Susceptibility to AITDs is initially attributed to major histocompatibility complex (MHC) class II genes. Subsequent research has identified numerous non-MHC genes that also play a role in the etiology of AITDs (22).

Graves’ disease and HT involve the production of autoantibodies—such as thyroid peroxidase antibodies (TPOAb), thyroglobulin antibody (TgAb), thyroid-stimulating antibody (TSAb), and thyroid-stimulating hormone (TSH)-receptor-blocking antibody (TBAb)—that target the thyroid gland, causing hormonal imbalances and potentially neurological symptoms. Both conditions are driven by T-cell responses and characterized by lymphocytic infiltration into the thyroid. Graves’ disease, a leading cause of hyperthyroidism in iodine-sufficient regions, is marked by excessive thyroid stimulation due to TSAb, leading to increased hormone release and gland hypertrophy. In contrast, HT is the most common autoimmune thyroiditis and predominantly results in hypothyroidism, primarily due to high levels of TPOAb and, to a lesser extent, TgAb (23).

This study aims to investigate the potential connection between gluten consumption and the development of autoimmune thyroid diseases.

## 2. Evidence Acquisition

### 2.1. Search Strategy

In this narrative review, the role of gluten in the development of AITDs was investigated by searching the electronic databases PubMed, Scopus, and Google Scholar for relevant studies published between 2000 and 2024. The following search terms were used: (“Gluten” OR “gluten-free” OR “gluten-free label” OR “gluten contamination” OR “gluten challenge” OR “gluten consumption” OR “gliadin” OR “prolamins” OR “hordein” OR “secalin” OR “glutenin”) AND (“celiac” OR “coeliac” OR “celiac disease” OR “CD” OR “CeD” OR “gluten enteropathy” OR “Gluten-Sensitive Enteropathy” OR “Nontropical Sprue” OR “Celiac Sprue”) AND ("Autoimmune thyroid disorders" OR "AITD" OR "Autoimmune thyroiditis" OR "Hashimoto thyroiditis" OR "Graves' disease" OR "Atrophic autoimmune thyroiditis"). Additional articles were identified by examining the reference lists of the included studies. Articles were then selected based on predefined inclusion and exclusion criteria.

### 2.2. Inclusion and Exclusion Criteria

All English-language studies that assessed the role of gluten in the development of AITDs were included. Studies were excluded if they lacked full text or were published in languages other than English.

## 3. Results

### 3.1. The Role of Gluten in Autoimmune Diseases Development

Gluten is implicated in the pathogenesis of autoimmune diseases through multiple mechanisms. One of the primary adverse effects of gluten is its immunogenicity, which is closely associated with the inflammatory response. Specifically, gluten, an immunogenic protein, stimulates the production of anti-gluten and anti-gliadin antibodies, even in non-celiac patients and healthy individuals. Moreover, it activates T lymphocytes, leading to the release of a variety of mediators and cytokines that contribute to tissue damage (24, 25).

Furthermore, gluten acts as a pro-oxidative agent, resulting in elevated levels of reactive oxygen species (ROS) and reactive nitrogen species (RNS). The oxidative balance is critical for cellular homeostasis, and such an imbalance is associated with the development of numerous chronic inflammatory and autoimmune disorders. The oxidative stress induced by gliadin has been extensively documented in various cell lines, including Caco-2, HT29, SH-SY5Y, T84, and LoVo (24). Increased levels of ROS interfere with the degradation of tTG2 by the ubiquitin-proteasome system, leading to an accumulation of tTG2 protein. This protein is involved in a range of biological processes, including signal transduction, cellular survival, apoptosis, and differentiation, thereby playing a significant role in autoimmune conditions such as CD (24, 25).

In addition to its roles in oxidative stress and immunogenicity, gluten peptides exhibit cytotoxic and apoptotic properties that can disrupt essential cellular metabolic networks. At a cellular level, gliadin has been shown to induce cytotoxicity, decrease cell viability and differentiation, promote the secretion of lactate dehydrogenase (LDH), trigger apoptotic pathways, and inhibit the synthesis of RNA, DNA, and glycoproteins in HCT116 cells (24).

Gluten also negatively impacts the microbiome, causing a decrease in microbial diversity and a shift in the metabolome toward an inflammatory state (24-26).

Moreover, gluten influences gene expression by modifying DNA methylation patterns, and the effects of gliadin on epigenetic regulation have been observed in cell lines associated with both CD and non-celiac disease, such as MH-SY5Y and Caco-2 (24).

### 3.2. The Role of Gluten in Autoimmune Thyroid Disorders Development

**Figure 1.**
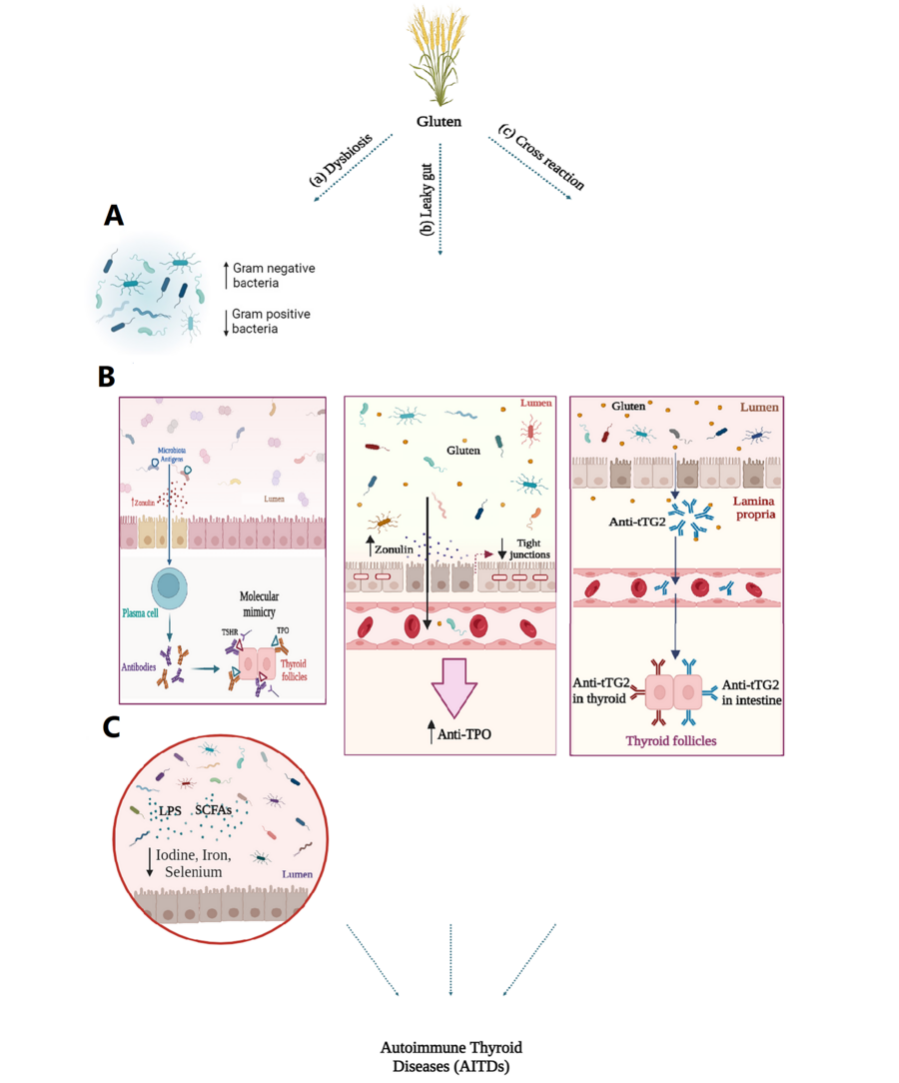
A, the roles of gluten in autoimmune thyroid disorders (AITDs) progression. In patients with AITDs, there is a decrease in gram-positive bacteria such as Firmicutes, Bifidobacteria, and Lactobacillus, and an increase in gram-negative bacteria such as Enterococcus; B, changes in gut microbiota composition lead to increased levels of zonulin and enhanced intestinal permeability. Consequently, microbiota antigens can activate the immune system through molecular mimicry. Structural similarities between thyroid autoantigens and bacterial antigens may result in antigenic mimicry, stimulating plasma cells to produce antibodies. As a result, autoreactive T cells are generated, triggering autoimmune responses; C, the microbiota influences the absorption of essential trace elements such as iodine, copper, iron, selenium, and zinc, which are crucial for thyroid health, through lipopolysaccharides (LPS) and short-chain fatty acids (SCFAs) released by the gut microbiota. Gluten consumption leads to the release of zonulin and disrupts the molecular integrity of tight junctions (TJs), which in susceptible individuals results in the development of leaky gut syndrome. Consequently, this allows antigens, toxins, and bacterial byproducts to enter the bloodstream, potentially influencing the development of AITDs and leading to increased levels of thyroid antibodies such as anti-TPO. Exposure to gluten and thyroid damage may occur through a molecular cross-reactivity mechanism between the gut and thyroid tissue transglutaminase 2 (tTG2). Anti-tTG2 antibodies present in the blood of individuals with CD may interact with tTG2 in the thyroid gland, potentially leading to the onset of thyroid disorders.

Gluten can play a role in the development and progression of AITDs through various mechanisms, including dysbiosis, leaky gut, and cross-reactivity, which will be explained in the following sections (Figure 1). 

#### 3.2.1. Autoimmune Thyroid Disorders and Gluten-Induced Dysbiosis

Previous studies have reported that maintaining gut health has systemic impacts on improving autoimmune disorder conditions (27). Maintaining gut health requires a complex interaction between the microbiota and the host immune system (27). Under normal conditions, the immune system and gut microbiota work together to coordinate the innate and adaptive immune responses (27).

In a 2020 study conducted by Mestre et al., it was reported that the consumption of probiotics by patients with multiple sclerosis resulted in a positive shift in the gut microbiome toward improved gut health, leading to enhanced mobility in these patients (28). In a 2017 study by Salehipour et al., probiotics were shown to reduce experimental autoimmune encephalomyelitis in mice by promoting CD4+ T-cell polarization toward regulatory T-cells, increasing anti-inflammatory cytokines, and inhibiting pro-inflammatory cytokines. This suppressed autoreactive T-cell proliferation and reduced leukocyte infiltration into the central nervous system (29).

Gluten serves as a modulator of human microbiota (30). It is proposed that the three-dimensional patterns formed by gliadin peptides could facilitate selective bacterial attachment and colonization, thereby disrupting gut homeostasis toward dysbiosis (30). Most studies indicate that dysbiosis in fecal and duodenal specimens of CD patients is marked by an increased presence of Gram-negative bacteria, such as Bacteroides and Enterobacteriaceae, and a reduced number of beneficial Gram-positive bacteria, like Bifidobacterium spp., when compared to healthy individuals (31). Nadal et al. found that small bowel biopsy samples from children with active CD had significantly higher proportions of total bacteria and Gram-negative bacteria compared to controls. Specifically, Lactobacillus and Bifidobacterium levels were significantly reduced, while Bacteroides and E. coli levels were significantly increased in children with active CD (32).

Research also indicates that gut microbiota compositions vary among healthy individuals and those with AITDs like HT, GD, and Graves' ophthalmopathy (GO), correlating with disease stage, thyroid autoantibody levels, and treatment response. Song et al. found that bacterial abundance and diversity were significantly reduced in patients with HT, especially those with hypothyroid status (HTH), compared to healthy controls. Lauritano et al. observed increased bacterial overgrowth in hypothyroid patients, while Zhou et al. noted a decrease in Bifidobacterium and Lactobacillus and an increase in Enterococcus in hyperthyroidism. Xie et al. found links between bacterial abundances and autoimmune thyroiditis parameters, such as anti-TG and anti-TPO antibodies. A meta-analysis revealed lower levels of Firmicutes, Bifidobacteria, and Lactobacillus in AITDs patients compared to controls. Hashimoto’s thyroiditis patients showed higher Bacteroides levels that were associated with altered host metabolism and autoantibody positivity (18, 21, 33-37).

Alterations in intestinal bacterial composition can increase intestinal permeability, which is associated with elevated levels of zonulin, a protein that regulates cell communication. When tight junctions between enterocytes are disrupted, antigens from the microbiota can penetrate and activate the immune system through molecular mimicry. The structural similarity between autoantigens and bacterial antigens in the colon may lead to antigenic mimicry that stimulates plasma cells to produce antibodies. Yersinia enterocolitica and Helicobacter pylori are considered environmental risk factors for GD, as their antigens exhibit cross-reactivity with self-antigens. This cross-reactivity, driven by structural or conformational similarities between microbial peptides and host antigens, can generate autoreactive T cells and trigger autoimmunity. For example, Yersinia enterocolitica porin proteins share sequence similarity with the thyrotropin receptor and can induce autoantibody production against the thyrotropin receptor. Additionally, IgG from Yersinia enterocolitica-infected patients can cause GD-like changes in thyroid structures, and GD patients have a higher infection rate of Yersinia enterocolitica compared to healthy controls. Helicobacter pylori also contributes to GD and GO, with its proteins, including NADH dehydrogenase subunit L and ABC transporter, partially aligning with the human thyrotropin receptor. The most virulent Helicobacter pylori strains, expressing cytotoxin-associated gene A antigens, are found more frequently in GD patients than in healthy individuals (38, 39).

Additionally, dysbiosis can lead to increased autoantibody synthesis through posttranslational protein modifications. Furthermore, it can promote the development of AITDs by shifting the Th1 lymphocyte pool to the Th2 subset and activating Toll-like receptor-4 (38).

Alterations in the composition of the microbiota have been linked to variations in TSH and TRAb levels. For example, it has been observed that levels of *Veillonella* and *Streptococcus* are inversely related to TSH and positively correlated with TRAb and TPOAb. *Veillonella* and *Streptococcus* also showed correlations with TGAb. Additionally, Bifidobacterium showed similar correlations. Strains of Lactobacillus and Bifidobacterium, due to their amino acid sequences resembling those of TG and TPO, may preferentially interact with autoantibodies, potentially triggering AITDs through molecular mimicry pathways (21, 38, 40).

The microbiome also plays a role in thyroid hormone metabolism, contributing to the gut-thyroid axis. Animal studies have demonstrated that intestinal bacteria can absorb deconjugated iodothyronine and compete with human albumin for binding thyroid hormones. Additionally, intestinal bacteria are involved in the enterohepatic metabolism of thyroid hormones (38).

Moreover, the microbiota influences the absorption of essential microelements such as iodine, copper, iron, selenium, and zinc, which are crucial for thyroid gland health. Studies in animals have shown that subjects deficient in microbiota exhibit reduced iodine absorption (38). In the human gastrointestinal tract, iodine uptake is mainly facilitated by the sodium-iodide symporter (NIS). Lipopolysaccharides and short-chain fatty acids released by the gut microbiota can influence iodine uptake by modulating the expression and activity of NIS (36).

Besides iodine, iron is essential for thyroid hormone synthesis, and iron deficiency is commonly associated with hypothyroidism. The gut microbiota can lower intestinal pH and increase the bioavailability of colonic iron through SCFA production, thereby affecting thyroid function (36). 

The thyroid contains selenium in the form of selenoproteins. Selenium-dependent enzymes such as glutathione peroxidase, deiodinases (D1, D2, and D3), and thioredoxin reductase maintain the stability and activity of thyroid hormones. The gut microbiota influences selenium metabolism and absorption in the colon (36).

#### 3.2.2. Autoimmune Thyroid Disorders and Gluten-Induced Leaky Gut

As stated before, exposure to gliadin disrupts the barrier integrity of intestinal epithelial cells. Multiple studies have shown that gliadin has the capacity to increase intestinal permeability and disrupt the molecular integrity of tight junctions (TJs), resulting in "leaky gut syndrome" in individuals predisposed to such effects (41-43). Leaky gut, characterized by enhanced intestinal permeability, permits antigens, toxins, and bacterial byproducts to enter the bloodstream from the gut, potentially influencing the development of AITDs (33, 44). The development of autoimmune diseases, including HT, has been associated with increased intestinal permeability (45).

Zonulin, a protein known to reversibly modulate TJ function, regulates intestinal permeability (46). Another potential physiological function of intestinal zonulin is to safeguard against the colonization of microorganisms in the proximal section of the intestine, thus contributing to innate immunity. Specifically, it plays a critical role in maintaining the balance between tolerance and immune response (46).

Gluten intake in the small intestine has been identified as a trigger for zonulin release. Clinical evidence supports the notion that zonulin upregulation may play a role in the development of HT (46). Özışık's research suggested a potential role for zonulin in the pathogenesis of HT, finding that serum zonulin levels were significantly elevated in patients compared to control groups (47). However, a 2022 study did not find a correlation between zonulin and TSH or anti-TG levels. Instead, a correlation was observed between zonulin and anti-TPO levels (48). In 2021, Zheng et al. reported elevated serum levels of biomarkers associated with leaky gut in GD patients. Specifically, levels of LPS, intestinal fatty acid-binding protein (I-FABP), zonulin, and D-lactate were significantly higher in patients with newly diagnosed GD compared to healthy controls. Logistic regression analysis indicated that zonulin and D-lactate were independently associated with the risk of GD, and circulating zonulin was found to effectively differentiate patients with newly diagnosed GD from healthy controls (43). In patients with HT, zonulin levels were found to be associated solely with the dose of levothyroxine after adjusting for age, weight, TSH, and fT4 levels (49). The mechanisms and effects of zonulin on AITDs have not yet been sufficiently investigated.

#### 3.2.3. Autoimmune Cross-Reactions Between CD and Autoimmune Thyroid Disorders

The interaction of antibodies targeting specific foods with diverse tissue antigens signifies possible cross-reactivity between food and human tissue antigens. The presence of these antibodies in the blood may potentially fuel the development of various autoimmune disorders (50).

The strongest connection between gluten exposure and thyroid destruction may be attributed to the molecular mimicry mechanism between gut and thyroid tTG (51). The concept of this molecular mimicry has been mentioned in studies, but detailed information in this regard is not yet available. It remains a topic of interest in the literature, and further research is required to confirm its role.

Tissue transglutaminase is a widely distributed enzyme found in all tissues, located both intra- and extracellularly. Due to the presence of tTG2 in tissues besides the intestines, autoimmune cross-reactions with other tissues (like the thyroid) may occur. It has been suggested that the anti-tTG2 antibodies found in the blood of individuals with CD might interact with tTG2 in the thyroid gland, potentially leading to the onset of thyroid disorders (52). This makes CD patients more susceptible to HT (45).

This hypothesis was confirmed in a study by Naiyer et al., where anti-tTG2 antibodies were found bound to follicular cells and the extracellular matrix of the thyroid through indirect staining. They also examined the impact of anti-tTG2 IgA antibodies on thyroid autoimmunity in individuals with CD. It was found that these antibodies bind to tTG2 present in thyroid tissue, indicating a potential link to the development of thyroid disorders in CD. Their research revealed that serum containing anti-tTG2 IgA from celiac patients binds to thyroid follicular cells (52).

A positive correlation was also observed between anti-tTG2 IgA and anti-TPO antibody levels, suggesting a connection between CD and the production of organ-specific antibodies targeting thyroid tissue (52). Marwaha et al. evaluated the presence of anti-tTG antibodies in patients with AITDs diagnosed by anti-TPO antibodies. Anti-tTG antibodies were at higher levels among cases compared to controls. The levels of anti-tTG antibodies increased with rising anti-TPO antibody levels (53). Hadizadeh Riseh et al. in their study reported that the prevalence of IgG and IgA anti-tTG antibodies and IgA anti-gliadin antibodies were higher in HT patients. There was a significant relationship between anti-thyroid antibodies and serum IgG-ATA and IgA anti-gliadin antibodies. In the ordinal regression model, serum IgG anti-tTG and IgA anti-gliadin antibodies were significant predictors of anti-thyroid antibodies in patients with HT and may play an important role in the exacerbation of disease progression (54). A study conducted in the United States showed a high prevalence of positive anti-tTG antibody titers in patients with AITDs, especially HT, in comparison to healthy individuals (55).

### 3.3. Gluten-Free Diet and Autoimmune Thyroid Disorders: A Review of Evidence

The primary objective of a GFD is to strictly eliminate gluten-containing food elements from the diet. The diet can be restrictive, as gluten is commonly used as a filler in processed foods and as an additive in products like malt. It is also utilized to enhance the texture and elasticity of baked goods, including cakes and bread (56).

A GFD is suggested to exert a beneficial effect on chronic autoimmune thyroiditis in patients with CD. Evidence indicates that a GFD may inhibit the autoimmune process by decreasing the concentration of thyroid antibodies and mitigating pathological changes in the small intestine (57). To date, only a limited number of studies have been conducted in this regard, and various reports have been published, as detailed below.

In 2024, Ulker et al. conducted a study involving 40 patients with HT. These patients were randomly assigned to one of four groups: Gluten-free, Mediterranean, Mediterranean gluten-free, and control. The participants were monitored over a 12-week period. The study found a statistically significant decrease in the levels of anti-TPO and anti-Tg antibodies across these groups following the GFD (58).

In a 2023 meta-analysis by Piticchio et al., an online search yielded 409 articles, of which 4 studies comprising 87 patients were included in the quantitative analysis. This meta-analysis demonstrated that a GFD over an average period of nearly 6 months resulted in a consistent decrease in TgAb and TPOAb titers in patients with HT (44).

In 2021, Poblocki et al. conducted a study involving 92 Caucasian women over 12 months. The study revealed that a GFD in patients with chronic autoimmune thyroiditis but without CD resulted in a reduction in TSH levels and an increase in free T4 levels compared to the control group. These findings suggest that a GFD may enhance the intestinal absorption of levothyroxine (59).

In 2019, Krysiak et al. conducted a study examining how a GFD affects the process of autoimmunization and thyroid gland function in individuals with HT. Participants were split into two groups: One adhering to a GFD and the other continuing with gluten consumption. Follow-up visits occurred every two months, during which blood concentrations of thyrotropin, free T3, anti-TPO, and anti-TG antibodies were assessed for each participant. Results indicated a decrease in anti-TPO and anti-TG antibody levels among patients adhering to a GFD (60).

However, since the impact of a GFD on AITDs is a relatively new topic, the sample sizes of studies addressing its significance vary greatly, with some being quite small. Larger studies or even clinical trials are needed in the future to confirm these data. Moreover, future studies should focus on healthy individuals and subjects with both CD and AITDs simultaneously. The long-term effects of a GFD should be examined, taking into account a broader range of factors and potential challenges, so that this diet can be recommended for AITDs with greater certainty (Table 1). 

**Table 1.** Studies About Effect of Gluten-Free Diet in Patients with Autoimmune Thyroid Disorders

Author Name	Age	Disease	GFD Duration	Hormone and Antibody Levels	Overall Effects of GFD
**Ulker et al. (** 58 **)**	18 - 65	HT	12-week	Increase in TSH, FT3 and FT4 hormone levels/ decrease in anti‐TPO and anti-Tg antibody levels	Effective against inflammation
**Piticchio et al. (** 44 **)**	25 - 42	HT	Nearly 6 months	Decrease in TgAb and TPOAb levels	Positive effect of the gluten deprivation on thyroid function and its inflammation
**Pobłocki et al. (** 59 **)**	18 - 55	Chronic autoimmune thyroid disease	Over 12 months	Decrease in anti-TPO and anti-TG antibody levels	Improves intestinal absorption of levothyroxine
**Krysiak et al. (** 60 **)**	20 - 45	AITD	2 months	Decrease in anti-TPO and anti-TG antibody levels	May bring clinical benefits to euthyroid women with Hashimoto’s thyroiditis

Abbreviations: GFD, gluten-free diet; HT, Hashimoto’s thyroiditis; AITD, autoimmune thyroid disorders.

## 4. Conclusions

In conclusion, it was determined that gluten, which plays a significant role in CD, influences the pathogenesis of AITDs in susceptible individuals through three mechanisms. Additionally, a GFD may contribute to the improvement of conditions in patients with AITDs. Although the positive effects of a GFD on AITDs have been observed in studies, these effects may be influenced by confounding factors such as dietary changes, age, adherence to the diet, and the strictness of dietary compliance, all of which can affect the outcomes.

Emerging evidence supports the need for personalized dietary strategies to mitigate risks associated with gluten intake. As we deepen our understanding of the gut-thyroid axis, this knowledge paves the way for innovative approaches in autoimmune care. Future studies should be conducted across a broader geographical area and with larger sample sizes to thoroughly examine the mechanisms underlying the relationship between gluten consumption and the onset of AITDs. Unknown mechanisms, such as the zonulin mechanism pathway and molecular mimicry, should also be investigated.

## Data Availability

The dataset presented in the study is available on request from the corresponding author during submission or after publication.
